# Roles of aminoacyl-tRNA synthetase-interacting multi-functional proteins in physiology and cancer

**DOI:** 10.1038/s41419-020-02794-2

**Published:** 2020-07-24

**Authors:** Zheng Zhou, Bao Sun, Shiqiong Huang, Dongsheng Yu, Xiaochuan Zhang

**Affiliations:** 1 Department of Chinese Medicine, The First Affiliated Hospital of Zhengzhou University, Zhengzhou, 450000 China; 2 Department of Clinical Pharmacology, Xiangya Hospital, Central South University, Changsha, 410000 China; 3 Hunan Key Laboratory of Pharmacogenetics, Institute of Clinical Pharmacology, Central South University, Changsha, 410000 China; 4 Department of Pharmacy, The First Hospital of Changsha, Changsha, 410005 China

**Keywords:** Cancer, Oncogenesis

## Abstract

Aminoacyl-tRNA synthetases (ARSs) are an important class of enzymes with an evolutionarily conserved mechanism for protein synthesis. In higher eukaryotic systems, eight ARSs and three ARS-interacting multi-functional proteins (AIMPs) form a multi-tRNA synthetase complex (MSC), which seems to contribute to cellular homeostasis. Of these, AIMPs are generally considered as non-enzyme factors, playing a scaffolding role during MSC assembly. Although the functions of AIMPs are not fully understood, increasing evidence indicates that these scaffold proteins usually exert tumor-suppressive activities. In addition, endothelial monocyte-activating polypeptide II (EMAP II), as a cleavage product of AIMP1, and AIMP2-DX2, as a splice variant of AIMP2 lacking exon 2, also have a pivotal role in regulating tumorigenesis. In this review, we summarize the biological functions of AIMP1, EMAP II, AIMP2, AIMP2-DX2, and AIMP3. Also, we systematically introduce their emerging roles in cancer, aiming to provide new ideas for the treatment of cancer.

## Facts

- AIMPs have various biological functions in addition to their roles as scaffolds in the MSC.
- AIMPs and their variants are related to the occurrence and development of cancer.
- Understanding of the molecular mechanisms linking AIMPs to cancer can contribute to identify new potential antitumoral strategies.

## Open questions

- Does the dysregulation of AIMPs in cancer affect the structure and function of the MSC?
- What is the molecular mechanism by which AIMPs regulate tumorigenesis?
- Is there potential for practical clinical applications based on findings concerning AIMPs in the context of cancer?

## Introduction

**Fig. 1:**
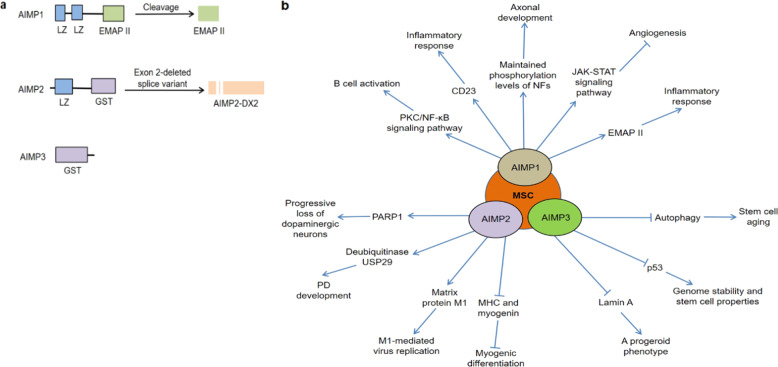
Structures and biological functions of AIMPs. **a** Major domains and variants of AIMPs. **b** Various biological functions of AIMPs.

Aminoacyl-tRNA synthetases (ARSs) are essential enzymes that participate in protein synthesis by catalyzing the activation of amino acids and linking them to their cognate transfer RNAs (tRNAs). In mammals, ARSs usually exist in free form or in the form of a multi-tRNA synthetase complex (MSC), and the latter consists of eight ARSs and three non-enzymatic ARS-interacting multi-functional proteins (AIMP1/p43, AIMP2/p38, and AIMP3/p18)^1^. Among them, AIMPs are generally considered as auxiliary proteins and play a role in scaffolding during MSC assembly^2^. In fact, AIMPs have several appended domains or motifs, which are involved in MSC formation and mediation of new functions^1^ (Fig. 1a). Meanwhile, AIMPs are closely linked with each other, and each of them has its preferable interacting ARSs in the MSC^3–6^. For example, X-ray crystallography confirmed that a ternary subcomplex consisting of aspartyl-tRNA synthetase (DRS), glutamyl-prolyl-tRNA synthetase (EPRS) and AIMP2 provided a key architecture in the MSC^7^. In this subcomplex, AIMP2 interacted with EPRS mainly through the heterodimerization of glutathione *S*-transferase (GST) domains, while DRS bound to AIMP2 by hydrogen bonds between the *α*7-*β*9 loop of DRS and the *β*2-*α*2 loop of AIMP2_GST_. Cho et al. found that four components, methionyl-tRNA synthetase (MRS), EPRS, AIMP2, and AIMP3, were assembled via a heterotetrameric complex structure of the GST domains in the human MSC^8^. Moreover, the full-length AIMP1 bound to AIMP3, and the N-terminus of AIMP1 bound to the GST-L domain of EPRS in the MSC^9^.

Interestingly, accumulating evidence has identified that AIMPs participate in various physiological and pathological processes as multifaceted molecules^10–13^. It was reported that the downregulation of AIMP1 enhanced transforming growth factor-β (TGF-β) signal by inducing the phosphorylation of Smad family member 2/3 (Smad2/3), which in turn promoted the chondrogenic potential of dedifferentiated/degenerated chondrocytes^14^. Furthermore, patients with a homozygous c.105C>A (p.Tyr35Ter) mutation in AIMP2 showed microcephaly, seizures, mental retardation, and spastic quadriplegia^15^. Recent studies found that AIMP3 could maintain genomic integrity through the DNA repair process, and the adult mice with AIMP3 deletion developed an acute radiation syndrome-like phenotype^16^. Strikingly, AIMPs are also related to tumorigenesis^17–19^, which suggested that studying the additional functions of AIMPs in the context of cancer could expand our understanding of tumorigenesis. Here, we not only summarize the biological functions of AIMP1, EMAP II, AIMP2, AIMP2-DX2, and AIMP3, but also focus on their emerging roles in regulating tumorigenesis, suggesting that their thorough study may provide new insights into cancer treatment.

## Biological functions of AIMPs

Apart from their role as scaffolds in the MSC, AIMPs also have a variety of biological functions^20–23^. Notably, these non-canonical functions are closely related to immune regulation, nervous system functions, angiogenesis, viral replication, and genome stability (Fig. 1b).

### AIMPs and immune regulation

AIMPs work as regulators or signaling molecules in some immune and inflammatory processes^24–26^. A previous study found that mammalian cells could specifically secrete the full-length AIMP1 without an apoptosis signal. In human monocytic THP-1 cells, AIMP1 could activate mitogen-activated protein kinase (MAPK) and nuclear factor-κB (NF-κB) and induce the expression of multiple cytokines as well as chemokines, such as tumor necrosis factor (TNF), interleukin 8 (IL-8), monocyte chemotactic protein 1 (MCP-1) and macrophage inflammatory protein-1α (MIP-1α), which were generally regarded as the main factors inducing atherosclerosis^27^. Importantly, AIMP1 was highly expressed in the foam cells of atherosclerotic lesions, indicating that it might promote the development of atherosclerosis by inducing cytokines and chemokines. CD23 (FcεRII) was thought to be a functional receptor molecule for AIMP1, which mediated the inflammatory response induced by AIMP1^28^. During this process, AIMP1 bound to CD23 and subsequently induced TNF-α secretion by activating extracellular signal-regulated kinase 1/2 (ERK1/2) in THP-1 cells and peripheral blood mononuclear cells (PBMCs). Notably, a neutralizing antibody MHM6 inhibited the secretion of TNF-α induced by AIMP1 via competing with IgE for binding to CD23, suggesting that the interaction between AIMP1 and CD23 in monocytes might play an important role in autoimmune diseases. Recently, Kim et al. demonstrated that AIMP1 inhibited T cell receptor (TCR)-mediated CD4 T cell activation and proliferation^29^. AIMP1 weakened the TCR signal by interfering with lipid raft aggregation and thus reduced the phosphorylation of phospholipase C γ (PLCγ) and phosphatidylinositol 3-hydroxy kinase (PI3K) in CD4 T cells. At the same time, AIMP1 specifically enhanced the differentiation of regulatory T (Treg) cells, but had no effect on the differentiation of T helper type 1 (Th1), Th2, and Th17 cells.

EMAP II was first discovered to be secreted by tumor cells and could induce acute inflammatory response in vivo^30^. Subsequent research showed that AIMP1 was the precursor of EMAP II, which was released after AIMP1 cleavage^31,32^. Lee et al. revealed that EMAP II promoted the development of bronchopulmonary dysplasia (BPD) in mice by recruiting macrophages to aggravate the inflammatory state^33^. By further confirming the transcription characteristics of macrophages exposed to EMAP II, researchers found that the unique transcription profile in response to EMAP II was mainly mediated by janus kinase (JAK)-signal transducer and activator of transcription 3 (STAT3), providing a direction for the study of EMAP II-mediated inflammation^34^. Furthermore, TCR affinity controlled the differentiation process by regulating the AIMP3, IL-2R α-chain (CD25) and guanylate binding protein 2 (Gbp2) in naive T cells^35^. Specifically, low TCR affinity induced the expression of Gbp2, thereby promoting T follicular helper (Tfh) cell differentiation, while high TCR affinity induced the expression of AIMP3 and CD25, thereby promoting non-Tfh cell formation.

### AIMPs and nervous system functions

Pathogenic mutations in AIMP1 have been reported to be associated with neurological diseases, such as neurodegenerative disease, pontocerebellar hypoplasia, and intellectual disability^36–38^. For example, a homozygous c.917A>G (p.Asp306Gly) mutation in AIMP1 caused severe neurodegenerative phenotypes, including developmental delays, epilepsy and progressive microcephaly^39^. Meanwhile, magnetic resonance imaging (MRI) showed that the neuroimaging features were callosal atrophy and T2 hyperintensity in the superficial white matter, as well as preserved myelination in the periventricular and deep white matter structures. Of note, AIMP1-deficient mice showed axon degeneration in motor neurons, defects of neuromuscular junctions, motor dysfunction and muscular atrophy^40^. AIMP1 was mainly expressed in central neurons and specifically interacted with the rod domain of neurofilament-light subunit (NF-L). Moreover, AIMP1 was a negative regulator of NF phosphorylation, and its overexpression or depletion could change the phosphorylation level of NFs, leading to the NF network disassembly. Recently, Xu et al. discovered that the N terminus of AIMP1 was responsible for the binding to its C terminus and arginyl-tRNA synthetase (RARS), and it also colocalized to the NF-L subunit protein^41^. These findings suggest that AIMP1 plays an important role in NF assembly and axon maintenance, which provides a new idea for exploring the pathogenesis of neurological diseases.

Interestingly, AIMP2 was a Parkin substrate^42^. In the dopaminergic neuroblastoma-derived SH-SY5Y cell line, Parkin promoted the ubiquitylation and degradation of AIMP2. Importantly, the overexpression of Parkin significantly protected SH-SY5Y cells from AIMP2-induced cell death. Lee et al. showed that AIMP2 accumulation overactivated poly(ADP-ribose) polymerase-1 (PARP1), resulting in the PAR accumulation and progressive loss of dopaminergic neurons, suggesting that AIMP2-induced parthanatos contributed to dopaminergic cell death, and that PARP1 inhibitors might be used to delay the progression of Parkinson’s disease (PD)^43^. Conspicuously, vacuolar protein sorting-associated protein 35 (VPS35) prevented AIMP2-mediated PARP1 activation and cell death by facilitating lysosomal degradation of AIMP2, suggesting that VPS35 might be a potential target for relieving PD^44^. AIMP2 transcriptionally upregulated deubiquitinase ubiquitin-specific peptidase 29 (USP29), resulting in the accumulation of 160 kDa myb-binding protein (MYBBP1A) in PD^45^. The expression of USP29 and MYBBP1A was upregulated in the Parkin knockout SH-SY5Y cells and the ventral midbrain of AIMP2 transgenic mice, suggesting that MYBBP1A might contribute to the development of PD.

### Other biological functions

A previous study by Park et al. demonstrated that AIMP1 played a dose-dependent biphasic role in angiogenesis^46^. Low concentrations of AIMP1 activated matrix metalloproteinase 9 (MMP9) through ERK, leading to the migration of endothelial cells. Conversely, high concentrations of AIMP1 activated c-Jun N-terminal kinase (JNK), thereby inducing the apoptosis of endothelial cells. Mechanistically, the JAK-STAT signaling pathway mediated the inhibitory effect of AIMP1 on angiogenesis^47^. Furthermore, many studies have shown that EMAP II is an effective antiangiogenic cytokine^48,49^. In myocardial infarction mice treated with EMAP II antibody, researchers found upregulated angiogenesis-related biomarkers and increased endothelial cell, suggesting that EMAP II blockade improved cardiac function by inducing angiogenesis^50^.

In influenza A virus (IAV)-infected cells, the virus NS2 protein protected host AIMP2 from ubiquitin-mediated degradation^51^. Subsequently, the accumulated AIMP2 promoted the conversion from ubiquitination to SUMOylation of matrix protein M1, thereby promoting M1-mediated virus replication. This finding suggests that AIMP2 plays an important role in IAV infection and may serve as a potential target for the treatment of influenza. Furthermore, researchers also discovered that AIMPs were associated with DNA damage. Liu et al. found that the rs12199241 of AIMP3 was significantly related to the levels of DNA damage in Chinese population^52^. Analogously, AIMP3 depletion in mouse embryonic stem cells blocked double-strand break repair, which resulted in the accumulation of DNA damage and genome instability^53^. In addition, AIMPs were also involved in other biological processes, such as glucose homeostasis, liver fibrosis, myogenic differentiation, and aging^54–58^.

## Roles of AIMPs in cancer

Cancer is a serious threat to human health and life and is rapidly becoming a major global health burden. A growing number of reports have described the association between AIMPs and cancer, suggesting the potential significance of AIMPs in cancer biology.

### AIMP1 and cancer

Lee et al. observed that AIMP1 had significant antitumor activity in a xenograft mouse model^59^. The expression of AIMP1, AIMP2, and AIMP3 was downregulated in gastric and colorectal cancer, which might result in their inactivation of tumor suppressor functions and tumor development^60^. Moreover, RARS could regulate the secretion of AIMP1 in HeLa and MCF7 cell lines^61^. In laryngeal squamous cell carcinoma (LSCC) tissues, AIMP1 and leukotriene A4 hydrolase (LTA4H) were upregulated and promoted the proliferation, migration, and invasion of LSCC cells^62^. Importantly, AIMP1 and LTA4H bound to fascin actin-bundling protein 1 (FSCN1), suggesting that their interaction might promote the progress of LSCC.

Particularly, AIMP1 plays an important regulatory role in tumor immunity. Based on the abundant immunophenotype in glioblastoma (GBM), Cheng et al. identified eight immune-related genes with prognostic value in GBM^63^. Of these, AIMP1, forkhead box O3 (FOXO3) and zinc finger and BTB domain containing 16 (ZBTB16) were defined as protective with HR < 1, whereas IL-6, IL-10, chemokine ligand 18 (CCL18), Fc fragment of IgG receptor IIb (FCGR2B) and MMP9 were defined as risky with HR > 1. Meaningfully, the researchers developed a local immune-related risk signature from these genes to distinguish cases as high or low risk of unfavorable prognosis.

**Fig. 2:**
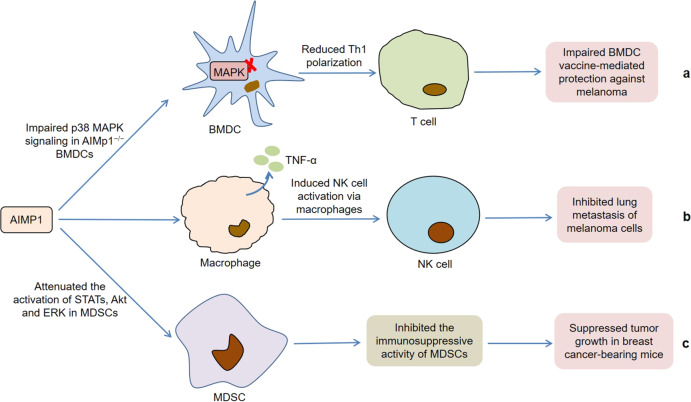
Roles of AIMP1 in tumor immunity. **a** The absence of AIMP1 in BMDCs reduces Th1 polarization of T cells by impairing p38 MAPK signaling, which significantly impairs BMDC vaccine-mediated protection against melanoma. **b** AIMP1 activates NK cells through the direct contact between macrophages and NK cells, which inhibits lung metastasis of melanoma cells. Meanwhile, AIMP1 also promotes the secretion of TNF-α by macrophages, which partially supports the activation of NK cells. **c** AIMP1 inhibits the immunosuppressive activity of MDSCs by attenuating the activation of STATs, Akt, and ERK, thereby suppressing the tumor growth in breast cancer-bearing mice.

Moreover, the absence of AIMP1 in bone marrow-derived dendritic cells (BMDCs) reduced downstream Th1 polarization of T cells by impairing p38 MAPK signaling, which significantly weakened BMDC vaccine-mediated protection against melanoma^64^. Meanwhile, The Cancer Genome Atlas (TCGA) database analysis showed that the expression of AIMP1 in nearly 9000 primary tumor samples was highly correlated with long-term survival. These results indicated that AIMP1 was critical for effective antitumor immunity. Kim et al. supported that AIMP1 activated natural killer (NK) cells through macrophages, which significantly inhibited lung metastasis of melanoma cells in vivo^65^. In this process, the direct contact between macrophages and NK cells was necessary for AIMP1-induced NK cell activation. AIMP1 also significantly promoted the secretion of TNF-α by macrophages, which partially supported the activation of NK cells. Interestingly, AIMP1 reduced the population of myeloid-derived suppressor cells (MDSCs) in the spleens and primary tumor sites of breast tumor-bearing mice and inhibited the expansion of MDSCs in tumor-conditioned media^66^. In addition, AIMP1 not only negatively regulated the inhibitory activity of MDSCs by reducing the production of IL-6, nitric oxide (NO), and arginase-1 (Arg-1), but also effectively attenuated the ability of MDSCs to suppress T cell proliferation and induce Treg cell differentiation in vivo. Further research discovered that the negative regulation of MDSC functions by AIMP1 might be related to the weakened activation of STATs, protein kinase B (Akt) and ERK. Collectively, these findings indicate that AIMP1 actively participates in tumor immunity through regulating various physiological processes, such as Th1 polarization, NK cell activity, and MDSC functions (Fig. 2).

### EMAP II and cancer

Previous research found that EMAP II was a tumor-suppressive cytokine with antiangiogenic effects, which inhibited the primary and metastatic tumor growth and facilitated apoptosis in growing capillary endothelial cells^67^. Low-dose EMAP II inhibited tumor growth by inducing defective autophagy and G2/M arrest in glioblastoma stem cells (GSCs)^68^. Mechanistically, EMAP II reduced the expression of phosphorylated PI3K and Akt with concomitant induction of FoxO1 activation. The specific knockout of FoxO1 greatly reduced the induction of autophagy and G2/M arrest, suggesting that the PI3K/Akt/FoxO1 axis was involved in the anticancer effect of EMAP II in GSCs. Li et al. demonstrated that EMAP II inhibited the viability, migration, and tube formation of GBM-induced endothelial cells (GECs) by inducing autophagy, thereby inhibiting GBM-induced angiogenesis^69^. GECs treated with EMAP II showed upregulated expression of microtubule-associated protein-1 light chain-3 (LC3) and sequestosome 1 (p62/SQSTM1) and blockage of PI3K/Akt/mammalian target of rapamycin (mTOR) pathway. At the mechanistic level, EMAP II downregulated the expression of miR-96, which upregulated the expression of LC3 and p62/SQSTM1 by directly targeting unfolded protein response (UPR)-related proteins, such as glucose-regulated protein 78 (GRP78), eukaryotic translation initiation factor 2 alpha (eIF2α) and C/EBP homologous protein (CHOP). Analogously, low-dose EMAP II induced autophagy by downregulating the expression of miR-20a in human U-87 and U-251 glioma cells^70^. In this process, miR-20a negatively regulated the expression of autophagy-related 5 (ATG5) and ATG7 by directly targeting their 3′-UTR, thereby activating the autophagy pathway.

In addition, EMAP II sensitized human melanoma to systemic TNF-α in vivo^71^. Mechanistic investigations found that the expression of TNF-R1 protein was not increased in human endothelial cells treated with EMAP II, but instead was redistributed from Golgi storage pools to cell membranes^72^. Meanwhile, EMAP II induced the membrane expression and mobilization of TNF-R1-associated death domain (TRADD) protein. Intriguingly, EMAP II was associated with the permeability of blood-tumor barrier (BTB)^73–75^. Liu et al. observed that EMAP II upregulated the expression of protein kinase C-α (PKC-α) and increased its activity by inhibiting the expression of miR-330-3p, resulting in a decreased expression of tight junction (TJ)-related proteins including zonulae occludens-1 (ZO-1), occludin and claudin-5, as well as an increased permeability of BTB^76^. MiR-429 also mediated the effects of EMAP II on the permeability of BTB^77^. Specifically, EMAP II significantly upregulated the expression of miR-429, which not only inhibited the expression of ZO-1 and occludin by directly interacting with them, but also reduced the expression of TJ-related proteins by downregulating the expression and phosphorylation of p70 ribosomal protein S6 kinase (p70S6K). Furthermore, caveolae-mediated transcellular pathway was involved in the increased permeability of BTB induced by EMAP II in C6 glioma rats^78^.

**Fig. 3:**
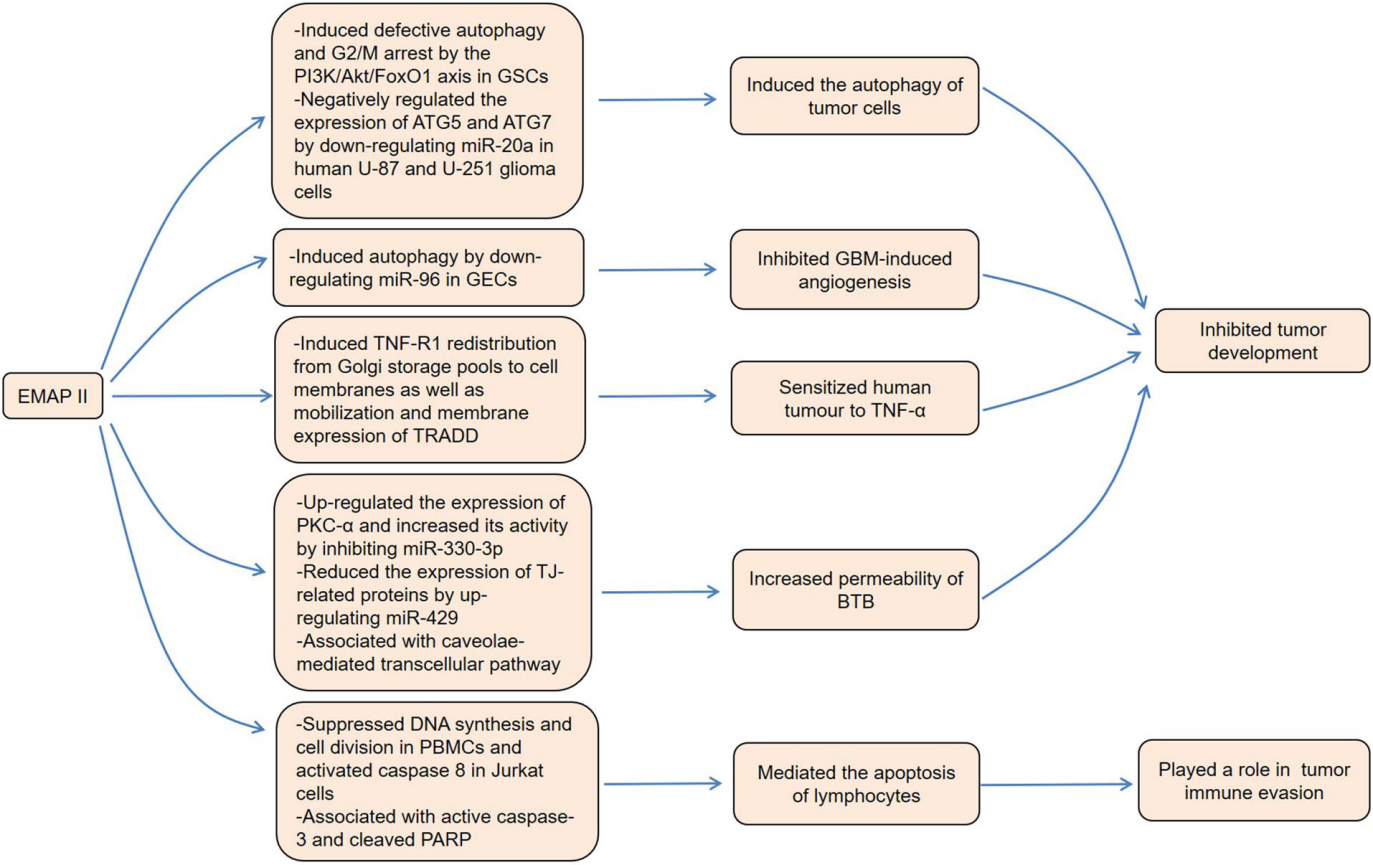
.Roles of EMAP II in the occurrence and development of cancer. On the one hand, EMAP II exerts antitumor effects by inducing tumorcell autophagy, inhibiting angiogenesis, sensitizing tumor cells to TNF-α, and increasing the permeability of BTB. On the other hand, EMAP II promotestumor development by inducing lymphocyte apoptosis.

Interestingly, in addition to the various antitumor effects described above, EMAP II is also associated with immune escape by tumor cells. Recombinant EMAP II suppressed DNA synthesis and cell division in PBMCs and induced apoptosis in PBMCs and Jurkat cells^79^. Consistently, native EMAP II expressed on the surface of HT29 cells could activate caspase 8 in Jurkat cells, which led to their apoptosis. Youssef et al. pointed out that colorectal cancer cells secreted EMAP II under hypoxic conditions, which mediated the apoptosis of tumor-infiltrating lymphocytes induced by hypoxia^80^. In conclusion, EMAP II not only exerts antitumor effects by inducing autophagy of tumor cells, inhibiting angiogenesis, sensitizing tumor cells to TNF-α, and increasing the permeability of BTB, but also promotes the development of cancer by inducing lymphocyte apoptosis (Fig. 3).

### AIMP2 and cancer

**Fig. 4:**
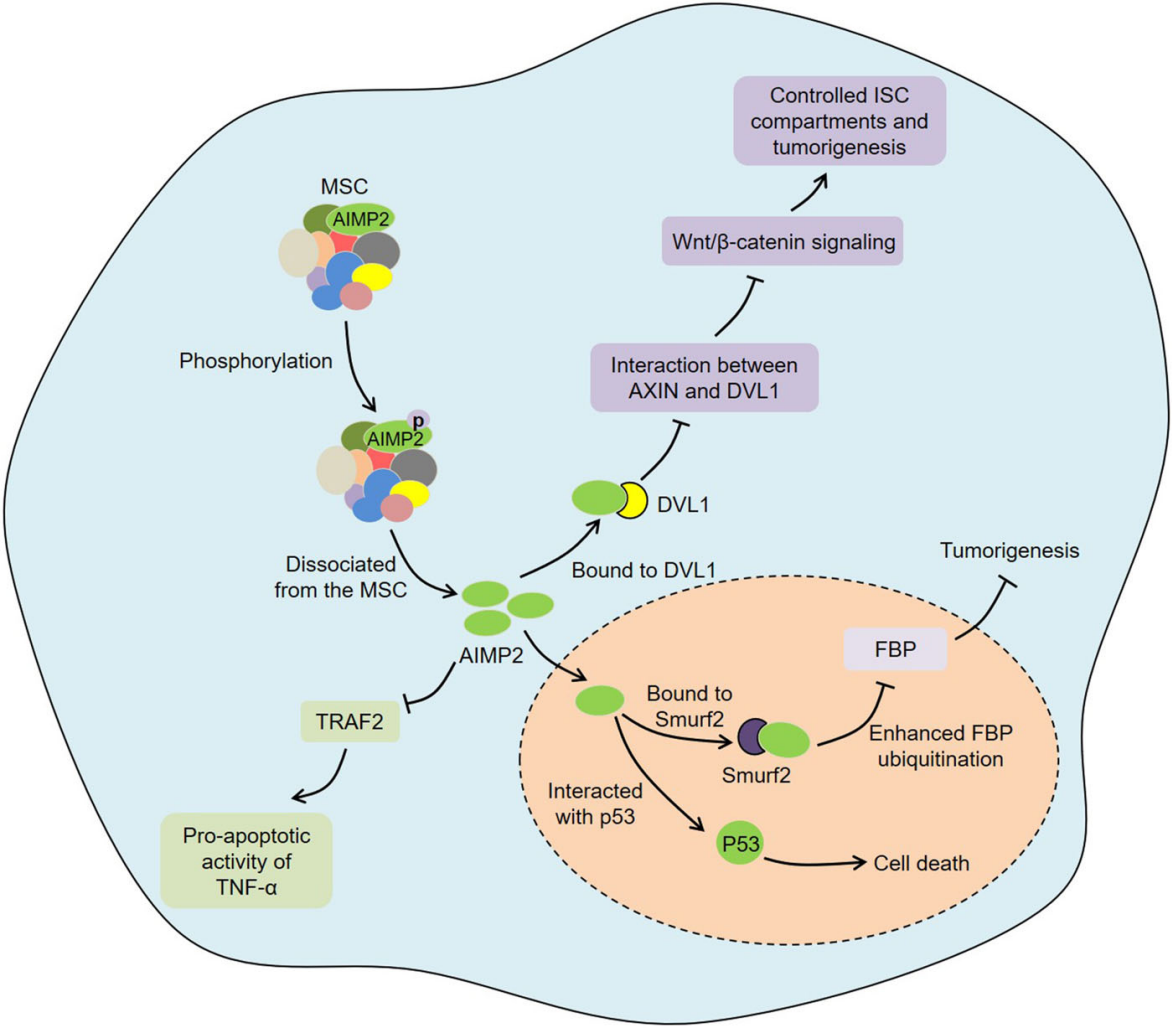
Roles of AIMP2 in cancer. Upon DNA damage, AIMP2 is phosphorylated and dissociates from the MSC. Subsequently, the dissociated AIMP2 translocates to the nucleus and directly interacts with tumor suppressor p53. AIMP2 promotes the pro-apoptotic activity of TNF-α via ubiquitin-mediated degradation of TRAF2. AIMP2 disrupts the interaction between AXIN and DVL1 by binding to DVL1, which inhibits Wnt/β-catenin signaling and therefore controls ISC compartments and tumorigenesis. Furthermore, the dissociated AIMP2 binds to Smurf2, thereby enhancing the ubiquitination of FBP and inhibiting tumorigenesis.

It is reported that AIMP2 usually shows tumor-suppressive activities (Fig. 4). A previous study by Choi et al. supported that the decreased AIMP2 levels in heterozygous AIMP2 mice provided greater sensitivity to multiple tumor formations, suggesting that AIMP2 could serve as a haploinsufficient tumor suppressor^81^. AIMP2-deficient cells were resistant to DNA damage-induced cell death, while the overexpression of AIMP2 enhanced the sensitivity to apoptosis^82^. Upon DNA damage, AIMP2 was phosphorylated by JNK and dissociated from the MSC. Subsequently, the dissociated AIMP2 translocated to the nucleus and directly interacted with tumor suppressor p53, thereby inhibiting murine double minute 2 (MDM2)-mediated ubiquitination and degradation of p53. These findings indicate that AIMP2 can regulate cell death through p53. It is well known that TNF-α is closely related to tumorigenesis^83,84^. TNF-α induced cell death was decreased in AIMP2-deficient cells^85^. Conversely, exogenous supplementation of AIMP2 enhanced the apoptotic sensitivity to TNF-α. Further studies shown that AIMP2 promoted the ubiquitin-dependent degradation of TNF receptor-associated factor 2 (TRAF2) by enhancing the recruitment of the E3 ligase c-IAP1 to TRAF2, thus mediating the pro-apoptotic activity of TNF-α. This finding suggests that AIMP2 may be involved in tumor development by regulating the TNF-α signaling pathway.

Hemizygous deletion of AIMP2 increased the formation of adenoma in Apc^Min/+^ mice and resulted in the proliferation of intestinal epithelial cells (IECs) in crypts and expansion of intestinal stem cell (ISC) compartments^86^. Further research found that AIMP2 disrupted the interaction between axis inhibition protein (AXIN) and Dishevelled-1 (DVL1) by binding to DVL1, which inhibited Wnt/β-catenin signaling and therefore controlled ISC compartments and tumorigenesis. Moreover, TGF-β induced AIMP2 expression and promoted its translocation to the nucleus, thereby participating in lung cell differentiation^87^. Mechanistically, the AIMP2 in the nucleus interacted with FUSE-binding protein (FBP), which stimulated the ubiquitination and degradation of FBP, leading to the downregulation of c-Myc. Another study discovered that upon TGF-β stimulation, AIMP2 was phosphorylated at S156 site by p38 MAPK and then dissociated from the MSC in HeLa cells^88^. The dissociated AIMP2 translocated to the nucleus and bound to Smad ubiquitin regulatory factor 2 (Smurf2), thereby enhancing the ubiquitination of FBP and inhibiting tumor formation. Notably, the S156A mutant in AIMP2 that inhibited its nuclear interaction with Smurf2 promoted tumorigenesis in vivo. Zhong et al. demonstrated that the RARS-mitotic arrest deficient-like 1 (MAD1L1) fusion protein interacted with AIMP2 to increase the expression of FBP, thereby promoting the occupation of the c-Myc promoter by FBP and subsequently inducing cancer stem cell (CSC)-like properties^89^. These data suggest that the development of drugs targeting the FBP/c-Myc axis via combinatorial therapy may be beneficial for certain types of cancer patients.

### AIMP2-DX2 and cancer

AIMP2-DX2, as a splice variant of AIMP2 lacking exon 2, was highly expressed in human lung cancer cells, and the ratio of AIMP2-DX2 to normal AIMP2 was increased with cancer progression^90^. Endogenous AIMP2-DX2 impaired the pro-apoptotic activity of AIMP2 through the competitive binding to p53 in lung cancer A549 cells. Interestingly, transgenic mice expressing AIMP2-DX2 showed increased sensitivity to lung tumorigenesis compared to the wild type counterpart. Jung et al. discovered that lung cancer patients with high AIMP2-DX2/AIMP2 autoantibody ratio had significantly shorter overall survival than those with low ratio, suggesting that AIMP2-DX2 levels were related to the clinical outcome of lung cancer^91^. Moreover, AIMP2-DX2 was highly expressed in chemoresistant ovarian cancer^92^. AIMP2-DX2 reduced the pro-apoptotic activity of TNF-α by competitively inhibiting the binding of AIMP2 to TRAF2, thereby contributing to the chemoresistance of ovarian cancer. Heat shock protein 70 (HSP70) was positively correlated with AIMP2-DX2 in lung cancer patient tissues^93^. Importantly, HSP70 could block the Seven in absentia homolog 1 (Siah1) binding and ubiquitination of AIMP2-DX2 by specifically recognizing and stabilizing AIMP2-DX2, leading to an increase in AIMP2-DX2 levels. X-ray crystallography and NMR analysis revealed that HSP70 bound to the amino (N)-terminal flexible region and glutathione S-transferase domain of AIMP2-DX2 through its substrate-binding domain. Furthermore, AIMP2-DX2 promoted the proliferation, migration, and invasion of nasopharyngeal carcinoma (NPC) cells by upregulating MMP-2 and MMP-9^94^. In short, AIMP2-DX2 can not only serve as a potential biomarker for lung cancer, but also participate in tumorigenesis. Interestingly, the downregulation of AIMP2-DX2 expression by shRNA suppressed the epidermal growth factor receptor (EGFR)/MAPK signaling pathway, thereby inhibiting glucose uptake and cancer cell growth^95^. Therefore, suppressing the expression of AIMP2-DX2 may be an effective strategy for treating cancer.

### AIMP3 and cancer

**Fig. 5:**
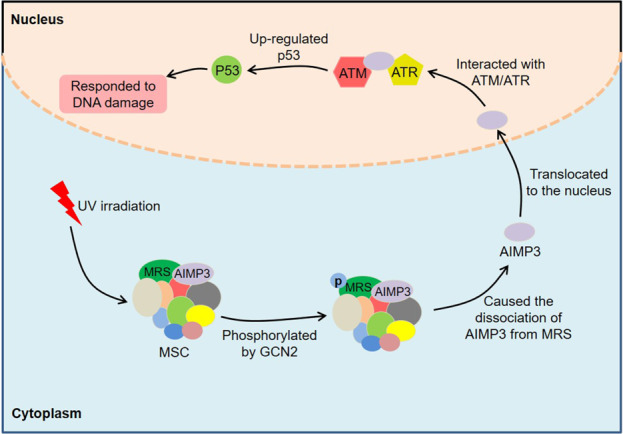
Roles of AIMP3 in cancer. Under UV irradiation, MRS is phosphorylated at Ser662 by GCN2, causing a conformational change in MRS and the subsequent dissociation of AIMP3 from MRS. The dissociated AIMP3 translocates to the nucleus and upregulates p53 by directly interacting with the FAT domains of ATM/ATR, thereby responding to DNA damage.

As with AIMP2, AIMP3 was also a haploinsufficient tumor suppressor^96^. AIMP3 upregulated p53 by directly interacting with the FAT domains of ataxia telangiectasia-mutated/ATM and Rad 3-related (ATM/ATR), thereby responding to DNA damage. Kim et al. observed several mutations of AIMP3 in chronic myeloid leukemia (CML) patients^97^. Of these, the mutations at Ser^87^, Val^98^, and Arg^144^ blocked the interaction between AIMP3 and ATM, suggesting that these residues had important functions for p53 activation. Generally, AIMP3 and MRS interacted through the GST-homology domains in the MSC^98^. Under UV irradiation, MRS was phosphorylated at Ser662 by general control nonrepressed-2 (GCN2), causing a conformational change in MRS and the subsequent dissociation of AIMP3 from MRS. The dissociated AIMP3 translocated to the nucleus, where it participated in the DNA damage response. Hepatitis B virus X protein (HBx) activated the lncRNA highly upregulated in liver cancer (HULC) promoter through cAMP-responsive element-binding protein (CREB), thereby upregulating HULC expression in liver cancer HepG2 cells^99^. It was worth noting that the upregulated HULC promoted the proliferation of liver cancer cells by downregulating AIMP3. These results indicate that AIMP3 works as an important signaling molecule in tumorigenesis (Fig. 5).

AIMP3 expression was reduced in muscle-invasive bladder cancer (MIBC), resulting in impaired Tp53 transactivity and genomic instability^100^. Significantly, the reduction of AIMP3 increased the resistance of cancer cells to ionizing radiation. At the same time, AIMP3 expression predicted relapse and overall survival after radiotherapy, indicating that it could serve as a potential clinical biomarker for MIBC. Intriguingly, higher AIMP3 expression was associated with better survival in gastric and colon adenocarcinoma, but with poor survival in breast, lung, and liver cancers^101^. Therefore, more in-depth research is needed to explore the relationship between AIMP3 and survival outcomes across cancer types.

## AIMPs and potential therapeutic interventions

Meaningfully, several studies have focused on the potential clinical applications of AIMPs in cancer treatment. After 6 days of intravenous injection of recombinant AIMP1 in a mouse xenograft model bearing human stomach cancer cells, the tumor volume and weight decreased significantly^102^. The cells exhibiting an active cell cycle progression were reduced in tumor tissues of AIMP1-treated mice, and the blood levels of TNF-α and IL-1β were increased, indicating that AIMP1 might play an anti-tumor role by inducing tumor inhibiting cytokines. Furthermore, pharmacokinetic studies of a single intravenous injection in rats found that AIMP1 showed a low clearance and a low volume of distribution, and its half-life was 6 min.

Interestingly, low-dose EMAP II induced Bcl-2/adenovirus E1B 19 kDa protein-interacting protein 3 (BNIP3)-mediated mitophagy by downregulating miR-24-3p, thereby enhancing the temozolomide cytotoxicity of GSCs^103^. Mice treated simultaneously with EMAP II, temozolomide and miR-24-3p inhibitor showed the smallest tumors and the longest survival rates, suggesting that the combined use of EMAP II and temozolomide might be a new approach for the treatment of glioma. Similarly, Awasthi et al. demonstrated that EMAP II enhanced the antitumor effects of sorafenib and gemcitabine in pancreatic ductal adenocarcinoma (PDAC)^104^. Compared to the control group, the combination therapy significantly improved animal survival. Notably, the serum EMAP II levels in patients with non-small cell lung cancer were significantly higher than in healthy subjects, and high serum EMAP II levels were associated with shorter survival, indicating that EMAP II could serve as a new biomarker for non-small cell lung cancer^105^.

In addition, many studies have attempted to suppress tumor development by targeting AIMP2-DX2^106,107^. Won et al. effectively inhibited the growth of cancer cells by designing a trans-splicing ribozyme that targeted and replaced the AIMP2-DX2 RNA with a new transcript^108^. This ribozyme performed the RNA replacement by a high-fidelity trans-splicing reaction with the targeted residue of AIMP2-DX2 RNA, but did not work on normal AIMP2 transcript. AIMP2-DX2 eliminated oncogene-induced cell death and aging by binding to and inhibiting p14/ARF, thereby promoting tumorigenesis^109^. Conspicuously, a novel compound, SLCB050, could reduce the viability of small cell lung cancer cells by inhibiting the interaction between AIMP2-DX2 and p14/ARF. Another inhibitor, BC-DXI-495, specifically bound to AIMP2-DX2 and blocked its interaction with HSP70, thus exerting an anti-tumor activity^93^. Furthermore, the label-free molecular probe based on G-quadruplex and strand displacement could sensitively and selectively detect AIMP2-DX2, which might be used for early diagnosis and monitoring the progression of relevant cancer^110^.

## Conclusion and future perspective

Since the MSC plays an important role in protein synthesis, it is important to understand its structural features and the physiological functions of its components. Of these, AIMPs are generally considered as auxiliary proteins and play a scaffolding role during MSC assembly. In addition, AIMPs also participate in a spectrum of biological processes, including immune regulation, nervous system functions, angiogenesis, and genome stability, which are considered unexpected because AIMPs are classified as housekeeping proteins. Interestingly, most of the non-canonical functions are more or less related to tumorigenesis. In fact, AIMPs and their variants do play a vital role in tumor biology (Table 1). Specifically, AIMPs usually exert tumor-suppressive activities, while AIMP2-DX2 is involved in the development of cancer. Furthermore, certain AIMPs are also regarded as biomarkers for cancer prognosis. Therefore, the study of AIMPs in the context of cancer will be a promising field. However, more molecular mechanisms are needed to further clarify the relationship between AIMPs and cancer.

**Table 1:** Roles of AIMPs in cancer.

AIMPs	Cancer type	Cell/tissue type	Effects	Mechanisms	References
AIMP1	LSCC	Hep2 and TU-177	Promoted the proliferation, migration and invasion of LSCC cells	AIMP1 and LTA4H were upregulated in LSCC tissues and interacted with FSCN1	^62^
	GBM	Tumor tissue samples were obtained from glioma patients	Defined as an immune-related gene with prognostic value in GBM		^63^
	Melanoma	B16F10 and B16F0-OVA	Impaired BMDC vaccine-mediated protection against melanoma	The absence of AIMP1 in BMDCs reduced downstream Th1 polarization by impairing p38 MAPK signaling	^64^
	Melanoma	B16F1	Inhibited lung metastasis of melanoma cells	Activated NK cells through macrophages	^65^
	Breast cancer	4T1	Suppressed tumor growth in breast cancer‑bearing mice	Negatively regulated MDSC functions by weakening the activation of STATs, Akt and ERK	^66^
	Stomach cancer	MKN45	The cells exhibiting an active cell cycle progression were reduced in AIMP1-treated mice	Induced tumor-suppressing cytokines, such as TNF-α and IL-1β	^102^
EMAP II	Lung cancer and breast cancer	LLC and MDA-MB 468	Inhibited the primary and metastatic tumor growth and facilitated apoptosis in growing capillary endothelial cells		^67^
	GBM	U87-MG	Inhibited tumor growth	Inducing defective autophagy and G2/M arrest in GSCs by PI3K/Akt/FoxO1	^68^
	GBM	U87	Inhibited GBM-induced angiogenesis	Induced autophagy by downregulating miR-96 in GECs	^69^
	GBM	U-87 and U-251	Inhibited the viability, migration and invasion of glioma cells	Negatively regulated the expression of ATG5 and ATG7 by downregulating miR-20a	^70^
	Melanoma	Pmel, 883, Smel and 1286	Sensitized human melanoma to TNF-α	Induced TNF-R1 redistribution from Golgi storage pools to cell membranes and mobilization and membrane expression of TRADD	^71,72^
	GBM	U87	Increased permeability of BTB	Upregulated the expression of PKC-α and increased its activity by inhibiting miR-330-3p	^76^
	GBM	U87	Increased permeability of BTB	Reduced the expression of TJ-related proteins by upregulating miR-429	^77^
	GBM	C6	Increased permeability of BTB	Associated with caveolae-mediated transcellular pathway	^78^
	Colorectal cancer	HT29, DLD-1, LS513 and HCT-15	Induced apoptosis in PBMCs and Jurkat cells	Suppressed DNA synthesis and cell division in PBMCs and activated caspase 8 in Jurkat cells	^79^
	Colorectal cancer	DLD-1 and HT29	Mediated the apoptosis of tumor-infiltrating lymphocytes induced by hypoxia	Associated with active caspase-3 and cleaved PARP	^80^
AIMP2		NCI-H157, A549 and NCI-H460	Functioned as a proapoptotic factor in response to DNA damage	Interacted with tumor suppressor p53	^82^
		HeLa	Mediated the pro-apoptotic activity of TNF-α	Promoted the ubiquitin-dependent degradation of TRAF2	^85^
	Colorectal cancer	HCT116 and HeLa	Controlled ISC compartments and tumorigenesis	Inhibited Wnt/β-catenin signaling	^86^
		A549	Participated in lung cell differentiation and suppressed proliferation of the epithelial carcinoma cells	Downregulated FBP and c-Myc	^87^
		WI-26, 293 T and HeLa	Inhibited tumor formation	Bound to Smurf2 and thus enhanced the ubiquitination of FBP	^88^
	NPC	CNE2, HK1 and S26	Induced CSC-like properties	RARS-MAD1L1 fusion protein interacted with AIMP2 to increase the expression of FBP	^89^
AIMP2-DX2	Lung cancer	A549, NCI-H460, H322 and H157	Increased susceptibility to carcinogen-induced lung tumorigenesis	AIMP2-DX2 impaired the pro-apoptotic activity of AIMP2 through binding to p53	^90^
	Ovarian cancer	A2780, SKOV3 and HeyA8	Contributed to the chemoresistance of ovarian cancer	Reduced the pro-apoptotic activity of TNF-α by competitively inhibiting the binding of AIMP2 to TRAF2	^92^
	Lung cancer	H522, H1435, H460, etc.	Led to an increase in AIMP2-DX2 levels	HSP70 blocked the Siah1 binding and ubiquitination of AIMP2-DX2	^93^
	NPC	5-8F, CNE-1 and CNE-2Z	Promoted the proliferation, migration and invasion of NPC cells	AIMP2-DX2 upregulated MMP-2 and MMP-9	^94^
	Lung cancer	H460	Inhibited the growth of cancer cells	Targeted and replaced the AIMP2-DX2 RNA with a new transcript by a trans-splicing ribozyme	^108^
	Lung cancer	NCI-H23, NCI-H322, NCI-H358 and NCI-H460	Reduced the viability of small cell lung cancer cells	SLCB050 inhibited the interaction between AIMP2-DX2 and p14/ARF	^109^
AIMP3		HCT116, A549 and H460	The AIMP3 heterozygous mice showed high susceptibility to tumors	Upregulated p53 by directly interacting with ATM/ATR, thereby responding to DNA damage	^96,97^
		HeLa	The dissociated AIMP3 translocated to the nucleus and participated in the DNA damage response	MRS was phosphorylated by GCN2, causing a conformational change in MRS and the subsequent dissociation of AIMP3 from MRS	^98^
	Liver cancer	HepG2, Hep3B and PLC/PRF/5	HULC promoted the proliferation of liver cancer cells	Downregulated the expression of AIMP3	^99^
	MIBC	T24, 253J, RT112 and RT4	The reduction of AIMP3 increased the resistance of cancer cells to ionizing radiation	Associated with impaired Tp53 transactivity and genomic instability	^100^

*AIMP1* ARS-interacting multi-functional protein 1, *TNF-α* tumor necrosis factor-alpha, *IL-1β* interleukin 1β, *RARS* arginyl-tRNA synthetase, *LSCC* laryngeal squamous cell carcinoma, *LTA4H* leukotriene A4 hydrolase, *FSCN1* fascin actin-bundling protein 1, *GBM* glioblastoma, *BMDC* bone marrow-derived dendritic cell, *MAPK* mitogen-activated protein kinase, *NK* natural killer, *MDSC* myeloid-derived suppressor cell, *STATs* signal transducers and activators of transcription, *Akt* protein kinase B, *ERK* extracellular signal-regulated kinase, *GSCs* glioblastoma stem cells, *PI3K* phosphatidylinositol 3-hydroxy kinase, *FoxO1* forkhead box O1, *GECs* GBM-induced endothelial cells, *ATG5* autophagy-related 5, *TRADD* TNF-R1-associated death domain, *BTB* blood-tumor barrier, *PKC-α* protein kinase C-α, *TJ* tight junction, *PBMCs* peripheral blood mononuclear cells, *PARP* Poly(ADP-ribose) polymerase, *TRAF2* TNF receptor associated factor 2, *ISC*, intestinal stem cell, *Smurf2* Smad ubiquitin regulatory factor 2, *FBP* FUSE-binding protein, *CSC* cancer stem cell, *MAD1L1* mitotic arrest deficient-like 1, *AIMP2-DX2* AIMP2 lacking exon 2, *HSP70* heat shock protein 70, *Siah1* seven in absentia homolog 1, *NPC* nasopharyngeal carcinoma, *MMP* matrix metalloproteinase, *ATM* ataxia telangiectasia-mutated, *ATR* ATM and Rad 3-related, *MRS* methionyl-tRNA synthetase, *GCN2* general control nonrepressed-2, *HULC* highly upregulated in liver cancer, *MIBC* muscle-invasive bladder cancer.

Indeed, as another component of the MSC, certain ARSs were also involved in tumorigenesis^111,112^. Genetic variants in several ARS genes have been reported to be associated with breast cancer risk in Chinese population^113^. Recently, Zirin et al. found that ARSs were important mediators of Myc growth control in *Drosophila*, and their inhibitors killed human cells overexpressing Myc, suggesting that ARSs might serve as targets for treating Myc-driven cancers^114^. AIMPs are also emerging as closely related to cancer biology, and taking them into consideration will lead to a better understanding of tumorigenesis and contribute to the treatment of malignant tumors.

## References

[1] Kwon, N. H., Fox, P. L. & Kim, S. Aminoacyl-tRNA synthetases as therapeutic targets. *Nat. Rev. Drug Discov.* **18**, 629–650 (2019). 10.1038/s41573-019-0026-3 31073243

[2] Rajendran, V., Kalita, P., Shukla, H., Kumar, A. & Tripathi, T. Aminoacyl-tRNA synthetases: Structure, function, and drug discovery. *Int. J. Biol. Macromol.* **111**, 400–414 (2018). 29305884 10.1016/j.ijbiomac.2017.12.157

[3] Guo, M., Ignatov, M., Musier-Forsyth, K., Schimmel, P. & Yang, X. L. Crystal structure of tetrameric form of human lysyl-tRNA synthetase: Implications for multisynthetase complex formation. *Proc. Natl Acad. Sci. USA* **105**, 2331–2336 (2008). 18272479 10.1073/pnas.0712072105 PMC2268136

[4] Fu, Y. et al. Structure of the ArgRS-GlnRS-AIMP1 complex and its implications for mammalian translation. *Proc. Natl Acad. Sci. USA* **111**, 15084–15089 (2014). 25288775 10.1073/pnas.1408836111 PMC4210331

[5] Cho, H. Y. et al. Symmetric assembly of a decameric subcomplex in human multi-tRNA synthetase complex via interactions between glutathione transferase-homology domains and aspartyl-tRNA synthetase. *J. Mol. Biol.* **431**, 4475–4496 (2019). 31473157 10.1016/j.jmb.2019.08.013

[6] Hei, Z., Wu, S., Liu, Z., Wang, J. & Fang, P. Retractile lysyl-tRNA synthetase-AIMP2 assembly in the human multi-aminoacyl-tRNA synthetase complex. *J. Biol. Chem.* **294**, 4775–4783 (2019). 10.1074/jbc.RA118.006356 PMC6442029 30733335

[7] Hahn, H. & Park, S. H. The DRS-AIMP2-EPRS subcomplex acts as a pivot in the multi-tRNA synthetase complex. *IUCrJ*. **6**, 958–967 (2019). 10.1107/S2052252519010790 PMC6760448 31576228

[8] Cho, H. Y. et al. Assembly of multi-tRNA synthetase complex via heterotetrameric glutathione transferase-homology domains. *J. Biol. Chem.* **290**, 29313–29328 (2015). 26472928 10.1074/jbc.M115.690867 PMC4705937

[9] Schwarz, M. A., Lee, D. D. & Bartlett, S. Aminoacyl tRNA synthetase complex interacting multifunctional protein 1 simultaneously binds Glutamyl-Prolyl-tRNA synthetase and scaffold protein aminoacyl tRNA synthetase complex interacting multifunctional protein 3 of the multi-tRNA synthetase complex. *Int. J. Biochem. Cell Biol.* **99**, 197–202 (2018). 29679766 10.1016/j.biocel.2018.04.015 PMC5959800

[10] Mirzaei, M. et al. Upregulation of proteolytic pathways and altered protein biosynthesis underlie retinal pathology in a mouse model of Alzheimer’s disease. *Mol. Neurobiol.* **56**, 6017–6034 (2019). 30707393 10.1007/s12035-019-1479-4

[11] Burgess, R. et al. The genetic landscape of epilepsy of infancy with migrating focal seizures. *Ann. Neurol.* **86**, 821-831 (2019). 10.1002/ana.25619 PMC7423163 31618474

[12] Kim, M. S., Kim, S. & Myung, H. Degradation of AIMP1/p43 induced by hepatitis C virus E2 leads to upregulation of TGF-beta signaling and increase in surface expression of gp96. *PLoS ONE* **9**, e96302 (2014). 24816397 10.1371/journal.pone.0096302 PMC4015952

[13] Kim, H. et al. Estrogen receptor activation contributes to RNF146 expression and neuroprotection in Parkinson’s disease models. *Oncotarget* **8**, 106721–106739 (2017). 29290984 10.18632/oncotarget.21828 PMC5739769

[14] Ahn, J. et al. AIMP1 downregulation restores chondrogenic characteristics of dedifferentiated/degenerated chondrocytes by enhancing TGF-beta signal. *Cell death Dis.* **7**, e2099 (2016). 26890138 10.1038/cddis.2016.17 PMC5399188

[15] Shukla, A. & Das Bhowmik, A. Homozygosity for a nonsense variant in AIMP2 is associated with a progressive neurodevelopmental disorder with microcephaly, seizures, and spastic quadriparesis. *J. Hum. Genet.* **63**, 19–25 (2018). 10.1038/s10038-017-0363-1 29215095

[16] Kim, D. et al. AIMP3 deletion induces acute radiation syndrome-like phenotype in mice. *Sci. Rep.* **8**, 15025 (2018). 30302025 10.1038/s41598-018-33303-3 PMC6177475

[17] Yin, K. et al. Using weighted gene co-expression network analysis to identify key modules and hub genes in tongue squamous cell carcinoma. *Medicine* **98**, e17100 (2019). 31517839 10.1097/MD.0000000000017100 PMC6750333

[18] Bronkhorst, I. H. et al. Effect of hypoxic stress on migration and characteristics of monocytes in uveal melanoma. *JAMA Ophthalmol.* **132**, 614–621 (2014). 24626595 10.1001/jamaophthalmol.2014.43

[19] Atala, A. Re: Loss of expression of the tumour suppressor gene AIMP3 predicts survival following radiotherapy in muscle-invasive bladder cancer. *J. Urol.* **194**, 1162–1163 (2015). 26382821 10.1016/j.juro.2015.07.013

[20] Kim, M. S. & Kim, T. S. Aminoacyl tRNA synthetase-interacting multifunctional protein 1 acts as a novel B cell-activating factor in vitro and in vivo. *J. Immunol. (Baltim., Md.: 1950)* **194**, 4729–4736 (2015). 10.4049/jimmunol.1401352 25870240

[21] Green, L. A. et al. HIV envelope protein gp120-induced apoptosis in lung microvascular endothelial cells by concerted upregulation of EMAP II and its receptor, CXCR3. *Am. J. Physiol. Lung Cell. Mol. Physiol.* **306**, L372–L382 (2014). 24318111 10.1152/ajplung.00193.2013 PMC3920224

[22] Kim, H. & Kang, S. J. Quantitative analysis of nasal transcripts reveals potential biomarkers for Parkinson’s disease. *Sci. Rep.* **9**, 11111 (2019). 10.1038/s41598-019-47579-6 PMC6668404 31366968

[23] Fan, X., Qi, B., Ma, L. & Ma, F. Screening of underlying genetic biomarkers for ankylosing spondylitis. *Mol. Med. Rep.* **19**, 5263–5274 (2019). 31059041 10.3892/mmr.2019.10188 PMC6522869

[24] Ahn, S. S., Kim, J. O., Yoon, T., Song, J. J. & Park, Y. B. Serum aminoacyl-tRNA synthetase-interacting multifunctional protein-1 can predict severe antineutrophil cytoplasmic antibody-associated vasculitis: a pilot monocentric study. *Biomed. Res. Int.* **2019**, 7508240 (2019). 10.1155/2019/7508240 PMC6545776 31236412

[25] Burastero, S. E. & Fabbri, M. Aminoacyl-tRNA synthetase-interacting multifunctional protein-1 (AIMP1): the member of a molecular hub with unexpected functions, including CD4 T cell homeostasis. *Clin. Immunol. (Orlando, Fla.)* **143**, 207–209 (2012). 10.1016/j.clim.2012.03.006 22542741

[26] Clauss, M. et al. Lung endothelial monocyte-activating protein 2 is a mediator of cigarette smoke-induced emphysema in mice. *J. Clin. Investig.* **121**, 2470–2479 (2011). 21576822 10.1172/JCI43881 PMC3104742

[27] Ko, Y. G. et al. A cofactor of tRNA synthetase, p43, is secreted to up-regulate proinflammatory genes. *J. Biol. Chem.* **276**, 23028–23033 (2001). 11292833 10.1074/jbc.M101544200

[28] Kwon, H. S. et al. Identification of CD23 as a functional receptor for the proinflammatory cytokine AIMP1/p43. *J. cell Sci.* **125**, 4620–4629 (2012). 22767513 10.1242/jcs.108209

[29] Kim, M. S., Lee, A., Cho, D. & Kim, T. S. AIMP1 regulates TCR signaling and induces differentiation of regulatory T cells by interfering with lipid raft association. *Biochem. Biophys. Res. Commun.* **514**, 875–880 (2019). 31084930 10.1016/j.bbrc.2019.05.040

[30] Kao, J. et al. Endothelial monocyte-activating polypeptide II. A novel tumor-derived polypeptide that activates host-response mechanisms. *J. Biol. Chem.* **267**, 20239–20247 (1992). 1400342

[31] Quevillon, S., Agou, F., Robinson, J. C. & Mirande, M. The p43 component of the mammalian multi-synthetase complex is likely to be the precursor of the endothelial monocyte-activating polypeptide II cytokine. *J. Biol. Chem.* **272**, 32573–32579 (1997). 9405472 10.1074/jbc.272.51.32573

[32] Shalak, V. et al. The EMAPII cytokine is released from the mammalian multisynthetase complex after cleavage of its p43/proEMAPII component. *J. Biol. Chem.* **276**, 23769–23776 (2001). 11306575 10.1074/jbc.M100489200

[33] Lee, D. D. et al. Endothelial monocyte-activating polypeptide II mediates macrophage migration in the development of hyperoxia-induced lung disease of prematurity. *Am. J. Respir. Cell Mol. Biol.* **55**, 602–612 (2016). 27254784 10.1165/rcmb.2016-0091OC PMC5070113

[34] Lee, D. D., Hochstetler, A., Murphy, C., Lowe, C. W. & Schwarz, M. A. A distinct transcriptional profile in response to endothelial monocyte activating polypeptide II is partially mediated by JAK-STAT3 in murine macrophages. *Am. J. Physiol. Cell Physiol.* **317**, C449–c456 (2019). 31216192 10.1152/ajpcell.00277.2018 PMC6766611

[35] Kotov, D. I., Mitchell, J. S. & Pengo, T. TCR affinity biases th cell differentiation by regulating CD25, Eef1e1, and Gbp2. *J Immunol.* **202**, 2535–2545 (2019). 30858199 10.4049/jimmunol.1801609 PMC6478541

[36] Accogli, A. et al. Biallelic loss-of-function variants in AIMP1 cause a rare neurodegenerative disease. *J. Child Neurol.* **34**, 74–80 (2019). 30486714 10.1177/0883073818811223

[37] Accogli, A. et al. Pathogenic variants in AIMP1 cause pontocerebellar hypoplasia. *Neurogenetics* **20**, 103–108 (2019). 30924036 10.1007/s10048-019-00572-7

[38] Iqbal, Z. et al. Missense variants in AIMP1 gene are implicated in autosomal recessive intellectual disability without neurodegeneration. *Eur. J. Hum. Genet.: EJHG* **24**, 392–399 (2016). 26173967 10.1038/ejhg.2015.148 PMC4755381

[39] BoAli, A. et al. Novel homozygous mutation of the AIMP1 Gene: a milder neuroimaging phenotype with preservation of the deep white matter. *Pediatr. Neurol.* **91**, 57–61 (2019). 30477741 10.1016/j.pediatrneurol.2018.09.010

[40] Zhu, X. et al. MSC p43 required for axonal development in motor neurons. *Proc. Natl Acad. Sci. USA* **106**, 15944–15949 (2009). 19717447 10.1073/pnas.0901872106 PMC2747223

[41] Xu, H., Malinin, N. L., Awasthi, N., Schwarz, R. E. & Schwarz, M. A. The N terminus of pro-endothelial monocyte-activating polypeptide II (EMAP II) regulates its binding with the C terminus, arginyl-tRNA synthetase, and neurofilament light protein. *J. Biol. Chem.* **290**, 9753–9766 (2015). 25724651 10.1074/jbc.M114.630533 PMC4392274

[42] Corti, O. et al. The p38 subunit of the aminoacyl-tRNA synthetase complex is a Parkin substrate: linking protein biosynthesis and neurodegeneration. *Hum. Mol. Genet.* **12**, 1427–1437 (2003). 12783850 10.1093/hmg/ddg159

[43] Lee, Y. et al. Parthanatos mediates AIMP2-activated age-dependent dopaminergic neuronal loss. *Nat. Neurosci.* **16**, 1392–1400 (2013). 23974709 10.1038/nn.3500 PMC3785563

[44] Yun, S. P. et al. VPS35 regulates parkin substrate AIMP2 toxicity by facilitating lysosomal clearance of AIMP2. *Cell Death Dis*. **8**, e2741 (2017). 10.1038/cddis.2017.157 PMC5477581 28383562

[45] Jo, A., Lee, Y., Park, C. H. & Shin, J. H. Deubiquitinase USP29 Governs MYBBP1A in the brains of Parkinson’s disease patients. *J. Clin. Med.*10.3390/jcm9010052 (2019). 10.3390/jcm9010052 PMC7019889 31878357

[46] Park, S. G. et al. Dose-dependent biphasic activity of tRNA synthetase-associating factor, p43, in angiogenesis. *J. Biol. Chem.* **277**, 45243–45248 (2002). 12237313 10.1074/jbc.M207934200

[47] Wang, W. et al. p43 induces IP-10 expression through the JAK-STAT signaling pathway in HMEC-1 cells. *Int. J. Mol. Med.* **38**, 1217–1224 (2016). 27574027 10.3892/ijmm.2016.2710

[48] Berger, A. C. et al. Endothelial monocyte activating polypeptide II induces endothelial cell apoptosis and may inhibit tumor angiogenesis. *Microvasc. Res.* **60**, 70–80 (2000). 10873516 10.1006/mvre.2000.2249

[49] Schwarz, M. A., Zheng, H., Liu, J., Corbett, S. & Schwarz, R. E. Endothelial-monocyte activating polypeptide II alters fibronectin based endothelial cell adhesion and matrix assembly via alpha5 beta1 integrin. *Exp. Cell Res.* **311**, 229–239 (2005). 16248999 10.1016/j.yexcr.2005.09.008

[50] Yuan, C. et al. Blockade of EMAP II protects cardiac function after chronic myocardial infarction by inducing angiogenesis. *J. Mol. Cell. Cardiol.* **79**, 224–231 (2015). 25456857 10.1016/j.yjmcc.2014.11.021 PMC4302026

[51] Gao, S. et al. Interaction of NS2 with AIMP2 facilitates the switch from ubiquitination to SUMOylation of M1 in influenza A virus-infected cells. *J. Virol.* **89**, 300–311 (2015). 25320310 10.1128/JVI.02170-14 PMC4301113

[52] Liu, J. et al. Genetic variants in multisynthetase complex genes are associated with DNA damage levels in Chinese populations. *Mutat. Res.* **786**, 8–13 (2016). 26871430 10.1016/j.mrfmmm.2016.01.006

[53] Kim, S. M., Jeon, Y., Kim, D. & Jang, H. AIMP3 depletion causes genome instability and loss of stemness in mouse embryonic stem cells. *Cell Death Dis*. **9**, 972 (2018). 10.1038/s41419-018-1037-4 PMC6155375 30250065

[54] Park, S. G. et al. Hormonal activity of AIMP1/p43 for glucose homeostasis. *Proc. Natl Acad. Sci. USA* **103**, 14913–14918 (2006). 17001013 10.1073/pnas.0602045103 PMC1595450

[55] Ahn, J. et al. Aminoacyl-tRNA synthetase interacting multi-functional protein 1 attenuates liver fibrosis by inhibiting TGFbeta signaling. *Int. J. Oncol.* **48**, 747–755 (2016). 26692190 10.3892/ijo.2015.3303

[56] Li, B. et al. MicroRNA-95 promotes myogenic differentiation by down-regulation of aminoacyl-tRNA synthase complex-interacting multifunctional protein 2. *Oncotarget* **8**, 111356–111368 (2017). 29340059 10.18632/oncotarget.22796 PMC5762327

[57] Kim, C., Park, J. M., Song, Y., Kim, S. & Moon, J. HIF1alpha-mediated AIMP3 suppression delays stem cell aging via the induction of autophagy. *Aging Cell* **18**, e12909 (2019). 30706629 10.1111/acel.12909 PMC6413650

[58] Oh, Y. S. et al. Downregulation of lamin A by tumor suppressor AIMP3/p18 leads to a progeroid phenotype in mice. *Aging Cell* **9**, 810–822 (2010). 20726853 10.1111/j.1474-9726.2010.00614.x

[59] Lee, Y. S. et al. Antitumor activity of the novel human cytokine AIMP1 in an in vivo tumor model. *Molecules Cells* **21**, 213–217 (2006). 16682815

[60] Kim, S. S., Hur, S. Y., Kim, Y. R., Yoo, N. J. & Lee, S. H. Expression of AIMP1, 2 and 3, the scaffolds for the multi-tRNA synthetase complex, is downregulated in gastric and colorectal cancer. *Tumori* **97**, 380–385 (2011). 21789020 10.1177/030089161109700321

[61] Bottoni, A. et al. Proteasomes and RARS modulate AIMP1/EMAP II secretion in human cancer cell lines. *J. Cell. Physiol.* **212**, 293–297 (2007). 17443684 10.1002/jcp.21083

[62] Gao, W. et al. Mass spectrometric analysis identifies AIMP1 and LTA4H as FSCN1-binding proteins in laryngeal squamous cell carcinoma. *Proteomics* **19**, e1900059 (2019). 10.1002/pmic.201900059 31287215

[63] Cheng, W. et al. Bioinformatic profiling identifies an immune-related risk signature for glioblastoma. *Neurology* **86**, 2226–2234 (2016). 27225222 10.1212/WNL.0000000000002770

[64] Liang, D. et al. AIMp1 potentiates TH1 polarization and is critical for effective antitumor and antiviral immunity. *Front. Immunol.* **8**, 1801 (2017). 29379495 10.3389/fimmu.2017.01801 PMC5775236

[65] Kim, M. S., Song, J. H., Cohen, E. P., Cho, D. & Kim, T. S. Aminoacyl tRNA synthetase–interacting multifunctional protein 1 activates NK cells via macrophages in vitro and in vivo. *J. Immunol. (Baltim., Md.: 1950)* **198**, 4140–4147 (2017). 10.4049/jimmunol.1601558 28381637

[66] Hong, H. J. et al. Aminoacyl-tRNA synthetase-interacting multifunctional protein 1 suppresses tumor growth in breast cancer-bearing mice by negatively regulating myeloid-derived suppressor cell functions. *Cancer Immunol. immunother.* **65**, 61–72 (2016). 26613952 10.1007/s00262-015-1777-2 PMC11029743

[67] Schwarz, M. A. et al. Endothelial-monocyte activating polypeptide II, a novel antitumor cytokine that suppresses primary and metastatic tumor growth and induces apoptosis in growing endothelial cells. *J. Exp. Med.* **190**, 341–354 (1999). 10430623 10.1084/jem.190.3.341 PMC2195582

[68] Liu, J. et al. Anti-neoplastic activity of low-dose endothelial-monocyte activating polypeptide-II results from defective autophagy and G2/M arrest mediated by PI3K/Akt/FoxO1 axis in human glioblastoma stem cells. *Biochemical Pharmacol.* **89**, 477–489 (2014). 10.1016/j.bcp.2014.04.014 24792437

[69] Li, Z. et al. Endothelial-monocyte activating polypeptide II suppresses the in vitro glioblastoma-induced angiogenesis by inducing autophagy. *Front. Mol. Neurosci.* **10**, 208 (2017). 28701921 10.3389/fnmol.2017.00208 PMC5488748

[70] Chen, J. et al. Low-dose endothelial-monocyte-activating polypeptide-II induced autophagy by down-regulating miR-20a in U-87 and U-251 glioma cells. *Front. Cell. Neurosci.* **10**, 128 (2016). 27242439 10.3389/fncel.2016.00128 PMC4868923

[71] Wu, P. C. et al. In vivo sensitivity of human melanoma to tumor necrosis factor (TNF)-alpha is determined by tumor production of the novel cytokine endothelial-monocyte activating polypeptide II (EMAPII). *Cancer Res.* **59**, 205–212 (1999). 9892208

[72] van Horssen, R., Rens, J. A., Schipper, D., Eggermont, A. M. & ten Hagen, T. L. EMAP-II facilitates TNF-R1 apoptotic signalling in endothelial cells and induces TRADD mobilization. *Apoptosis* **11**, 2137–2145 (2006). 17051333 10.1007/s10495-006-0284-5

[73] Xie, H., Xue, Y. X., Liu, L. B. & Liu, Y. H. Endothelial-monocyte-activating polypeptide II increases blood-tumor barrier permeability by down-regulating the expression levels of tight junction associated proteins. *Brain Res.* **1319**, 13–20 (2010). 20083091 10.1016/j.brainres.2010.01.023

[74] Li, Z. et al. Roles of serine/threonine phosphatases in low-dose endothelial monocyte-activating polypeptide-II-induced opening of blood-tumor barrier. *J. Mol. Neurosci.* **57**, 11–20 (2015). 26087743 10.1007/s12031-015-0604-8

[75] Li, Z. et al. Low-dose endothelial monocyte-activating polypeptide-II increases permeability of blood-tumor barrier via a PKC-zeta/PP2A-dependent signaling mechanism. *Exp. cell Res.* **331**, 257–266 (2015). 25592443 10.1016/j.yexcr.2014.12.021

[76] Liu, J. et al. The role of miR-330-3p/PKC-alpha signaling pathway in low-dose endothelial-monocyte activating polypeptide-II increasing the permeability of blood-tumor barrier. *Front. Cell. Neurosci.* **11**, 358 (2017). 29311822 10.3389/fncel.2017.00358 PMC5742213

[77] Chen, L. et al. MiR-429 regulated by endothelial monocyte activating polypeptide-II (EMAP-II) influences blood-tumor barrier permeability by inhibiting the expressions of ZO-1, occludin and claudin-5. *Front. Mol. Neurosci.* **11**, 35 (2018). 29467620 10.3389/fnmol.2018.00035 PMC5808301

[78] Li, Z., Liu, Y. H., Xue, Y. X., Liu, L. B. & Wang, P. Low-dose endothelial monocyte-activating polypeptide-ii increases permeability of blood-tumor barrier by caveolae-mediated transcellular pathway. *J. Mol. Neurosci.* **52**, 313–322 (2014). 24526454 10.1007/s12031-013-0148-8

[79] Murray, J. C. et al. Colorectal cancer cells induce lymphocyte apoptosis by an endothelial monocyte-activating polypeptide-II-dependent mechanism. *J. Immunol. (Baltim., Md.: 1950)* **172**, 274–281 (2004). 10.4049/jimmunol.172.1.274 14688335

[80] Youssef, M. M., Symonds, P., Ellis, I. O. & Murray, J. C. EMAP-II-dependent lymphocyte killing is associated with hypoxia in colorectal cancer. *Br. J. Cancer* **95**, 735–743 (2006). 16929248 10.1038/sj.bjc.6603299 PMC2360520

[81] Choi, J. W., Um, J. Y., Kundu, J. K., Surh, Y. J. & Kim, S. Multidirectional tumor-suppressive activity of AIMP2/p38 and the enhanced susceptibility of AIMP2 heterozygous mice to carcinogenesis. *Carcinogenesis* **30**, 1638–1644 (2009). 19622630 10.1093/carcin/bgp170

[82] Han, J. M. et al. AIMP2/p38, the scaffold for the multi-tRNA synthetase complex, responds to genotoxic stresses via p53. *Proc. Natl Acad. Sci. USA* **105**, 11206–11211 (2008). 18695251 10.1073/pnas.0800297105 PMC2516205

[83] Cruceriu, D., Baldasici, O. & Balacescu, O. The dual role of tumor necrosis factor-alpha (TNF-alpha) in breast cancer: molecular insights and therapeutic approaches. *Cell Oncol (Dordr)*. **43**, 1–18 (2020). 10.1007/s13402-019-00489-1 PMC12990688 31900901

[84] Lee, E. et al. The pleiotropic effects of TNFalpha in breast cancer subtypes is regulated by TNFAIP3/A20. *Oncogene* **38**, 469–482 (2019). 10.1038/s41388-018-0472-0 PMC6602794 30166590

[85] Choi, J. W. et al. AIMP2 promotes TNFalpha-dependent apoptosis via ubiquitin-mediated degradation of TRAF2. *J. Cell Sci.* **122**, 2710–2715 (2009). 19584093 10.1242/jcs.049767

[86] Yum, M. K. et al. AIMP2 controls intestinal stem cell compartments and tumorigenesis by modulating wnt/beta-catenin signaling. *Cancer Res.* **76**, 4559–4568 (2016). 27262173 10.1158/0008-5472.CAN-15-3357

[87] Kim, M. J. et al. Downregulation of FUSE-binding protein and c-myc by tRNA synthetase cofactor p38 is required for lung cell differentiation. *Nat. Genet.* **34**, 330–336 (2003). 12819782 10.1038/ng1182

[88] Kim, D. G. et al. Oncogenic mutation of AIMP2/p38 inhibits its tumor-suppressive interaction with Smurf2. *Cancer Res.* **76**, 3422–3436 (2016). 27197155 10.1158/0008-5472.CAN-15-3255

[89] Zhong, Q. et al. The RARS-MAD1L1 fusion gene induces cancer stem cell-like properties and therapeutic resistance in nasopharyngeal carcinoma. *Clin. Cancer Res.* **24**, 659–673 (2018). 29133573 10.1158/1078-0432.CCR-17-0352 PMC5796860

[90] Choi, J. W. et al. Cancer-associated splicing variant of tumor suppressor AIMP2/p38: pathological implication in tumorigenesis. *PLoS Genet.* **7**, e1001351 (2011). 21483803 10.1371/journal.pgen.1001351 PMC3069106

[91] Jung, J. Y. et al. Ratio of autoantibodies of tumor suppressor AIMP2 and Its oncogenic variant is associated with clinical outcome in lung cancer. *J. Cancer* **8**, 1347–1354 (2017). 28638448 10.7150/jca.18450 PMC5479239

[92] Choi, J. W. et al. Splicing variant of AIMP2 as an effective target against chemoresistant ovarian cancer. *J. Mol. cell Biol.* **4**, 164–173 (2012). 22532625 10.1093/jmcb/mjs018

[93] Lim, S. et al. Targeting the interaction of AIMP2-DX2 with HSP70 suppresses cancer development. *Nat. Chem. Biol.* **16**, 31–41 (2020). 10.1038/s41589-019-0415-2 31792442

[94] Cao, Q., Zhang, J. & Zhang, T. AIMP2-DX2 promotes the proliferation, migration, and invasion of nasopharyngeal carcinoma cells. *Biomed. Res. Int.* **2018**, 9253036 (2018). 10.1155/2018/9253036 PMC5941793 29854811

[95] Chang, S. H. et al. Lentiviral vector-mediated shRNA against AIMP2-DX2 suppresses lung cancer cell growth through blocking glucose uptake. *Mol. Cells* **33**, 553–562 (2012). 22562359 10.1007/s10059-012-2269-2 PMC3887752

[96] Park, B. J. et al. The haploinsufficient tumor suppressor p18 upregulates p53 via interactions with ATM/ATR. *Cell* **120**, 209–221 (2005). 15680327 10.1016/j.cell.2004.11.054

[97] Kim, K. J. et al. Determination of three-dimensional structure and residues of the novel tumor suppressor AIMP3/p18 required for the interaction with ATM. *J. Biol. Chem.* **283**, 14032–14040 (2008). 18343821 10.1074/jbc.M800859200

[98] Kwon, N. H. et al. Dual role of methionyl-tRNA synthetase in the regulation of translation and tumor suppressor activity of aminoacyl-tRNA synthetase-interacting multifunctional protein-3. *Proc. Natl Acad. Sci. USA* **108**, 19635–19640 (2011). 22106287 10.1073/pnas.1103922108 PMC3241768

[99] Du, Y. et al. Elevation of highly up-regulated in liver cancer (HULC) by hepatitis B virus X protein promotes hepatoma cell proliferation via down-regulating p18. *J. Biol. Chem.* **287**, 26302–26311 (2012). 22685290 10.1074/jbc.M112.342113 PMC3406714

[100] Gurung, P. M. et al. Loss of expression of the tumour suppressor gene AIMP3 predicts survival following radiotherapy in muscle-invasive bladder cancer. *Int. J. cancer* **136**, 709–720 (2015). 24917520 10.1002/ijc.29022

[101] Hassan, M. K., Kumar, D., Naik, M. & Dixit, M. The expression profile and prognostic significance of eukaryotic translation elongation factors in different cancers. *PLoS ONE* **13**, e0191377 (2018). 10.1371/journal.pone.0191377 PMC5771626 29342219

[102] Han, J. M., Myung, H. & Kim, S. Antitumor activity and pharmacokinetic properties of ARS-interacting multi-functional protein 1 (AIMP1/p43). *Cancer Lett.* **287**, 157–164 (2010). 19573982 10.1016/j.canlet.2009.06.005

[103] Zhang, J. et al. Endothelial monocyte-activating polypeptide-II induces BNIP3-mediated mitophagy to enhance temozolomide cytotoxicity of glioma stem cells via down-regulating MiR-24-3p. *Front. Mol. Neurosci.* **11**, 92 (2018). 29632473 10.3389/fnmol.2018.00092 PMC5879952

[104] Awasthi, N., Zhang, C., Hinz, S., Schwarz, M. A. & Schwarz, R. E. Enhancing sorafenib-mediated sensitization to gemcitabine in experimental pancreatic cancer through EMAP II. *J. Exp. Clin. Cancer Res.* **32**, 12 (2013). 23497499 10.1186/1756-9966-32-12 PMC3618297

[105] Sen, E., Ulger, F., Kaya, A., Akar, N. & Gonullu, U. Serum endothelial monocyte-activating polypeptide-II: a novel biomarker in patients with non-small-cell lung cancer. *Clin. lung cancer* **9**, 166–170 (2008). 18621627 10.3816/CLC.2008.n.025

[106] Lee, H. S. et al. Chemical suppression of an oncogenic splicing variant of AIMP2 induces tumour regression. *Biochemical J.* **454**, 411–416 (2013). 10.1042/BJ20130550 23815603

[107] Hwang, S. K., Chang, S. H., Minai-Tehrani, A., Kim, Y. S. & Cho, M. H. Lentivirus-AIMP2-DX2 shRNA suppresses cell proliferation by regulating Akt1 signaling pathway in the lungs of AIMP2(+)/(−) mice. *J. Aerosol Med. Pulm. Drug Deliv.* **26**, 165–173 (2013). 23517169 10.1089/jamp.2011.0959

[108] Won, Y. S. & Lee, S. W. Selective regression of cancer cells expressing a splicing variant of AIMP2 through targeted RNA replacement by trans-splicing ribozyme. *J. Biotechnol.* **158**, 44–49 (2012). 22285955 10.1016/j.jbiotec.2012.01.006

[109] Oh, A. Y. et al. Inhibiting DX2-p14/ARF interaction exerts antitumor effects in lung cancer and delays tumor progression. *Cancer Res.* **76**, 4791–4804 (2016). 27302160 10.1158/0008-5472.CAN-15-1025

[110] Zang, R., Wang, X., Zhu, Y., Yao, T. & Shi, S. Label-free molecular probe based on G-quadruplex and strand displacement for sensitive and selective detection and naked eye discrimination of exon 2 deletion of AIMP2. *Chem Biol Drug Des.* **93**, 993–998 (2019). 10.1111/cbdd.13406 30345633

[111] Lee, J. M. & Kim, T. Methionyl-tRNA synthetase is a useful diagnostic marker for lymph node metastasis in non-small cell lung cancer. *Yonsei. Med. J.* **60**, 1005–1012 (2019). 10.3349/ymj.2019.60.11.1005 PMC6813140 31637881

[112] Kim, S. B., Kim, H. R., Park, M. C., Cho, S. & Goughnour, P. C. Caspase-8 controls the secretion of inflammatory lysyl-tRNA synthetase in exosomes from cancer cells. *J. Cell Biol.* **216**, 2201–2216 (2017). 10.1083/jcb.201605118 PMC5496609 28611052

[113] He, Y. et al. Potentially functional polymorphisms in aminoacyl-tRNA synthetases genes are associated with breast cancer risk in a Chinese population. *Mol. Carcinog.* **54**, 577–583 (2015). 24510587 10.1002/mc.22128

[114] Zirin, J. et al. Interspecies analysis of MYC targets identifies tRNA synthetases as mediators of growth and survival in MYC-overexpressing cells. *Proc Natl Acad Sci USA* **116**, 14614–14619 (2019). 10.1073/pnas.1821863116 PMC6642371 31262815

